# The Prevalence and the Underlying Mechanisms of Fosfomycin Resistance of *Escherichia coli* and *Salmonella* spp. Among Cattle in Japan

**DOI:** 10.3390/ijms252413723

**Published:** 2024-12-23

**Authors:** Yuta Hosoi, Michiko Kawanishi, Saki Harada, Mio Kumakawa, Mari Matsuda, Hideto Sekiguchi

**Affiliations:** Veterinary AMR Center, National Veterinary Assay Laboratory, Ministry of Agriculture, Forestry and Fisheries, Tokyo 185-8511, Japan

**Keywords:** antimicrobial resistance, fosfomycin, whole-genome sequencing, antimicrobial susceptibility testing, *E. coli*, *Salmonella*

## Abstract

To investigate fosfomycin resistance rates in cattle across Japan, we carried out susceptibility tests. To identify the genes contributing to fosfomycin resistance, we performed whole-genome sequencing on the fosfomycin-resistant strains. *Escherichia coli* were sampled from healthy cattle (*n* = 292, combined total from 2017, 2020, 2021, and 2022) and diseased cattle (*n* = 73, from 2021 to 2022). *Salmonella* spp. were obtained from diseased cattle (*n* = 74 from 2021 to 2022). These samples originated from different and non-duplicated farms. The MICs to fosfomycin were measured using an agar dilution method with a breakpoint of 256 μg/mL. We conducted whole-genome sequencing with a MiSeq, followed by in silico analysis of the acquired draft genomes. The resistance rates were 0.3% (95% CI [0–1.9%]), 6.8% (95% CI [2.3–15.3%]), and 1.4% (95% CI [0–7.3%]). The *FosA3* gene was detected in five out of six fosfomycin-resistant *E. coli* strains and one *Salmonella* spp. strain. The fosfomycin-resistant *Salmonella* spp. strain also has a *fosA7* gene. One *E. coli* strain showed resistance to fosfomycin without having the *fosA3* gene, and with the mutations of *glpT*, *uhpT*, *uhpT* and *ptsI*, and with the existence of efflux pumps. The nationwide scale of resistance rates to fosfomycin in *E. coli* isolated from healthy and diseased cattle and that of *Salmonella* spp. from diseased cattle were revealed for the first time, and the resistance rates were low. In addition, genes linked to the mechanism of fosfomycin resistance were identified.

## 1. Introduction

Fosfomycin has been categorized as one of the highest-priority critically important antimicrobials (HPCIAs) on the WHO’s list of medically important antimicrobials since February 2024. It is reported that there is evidence of the emergence and dissemination of plasmid-mediated fosfomycin resistance genes in food-producing animals [1]. In Japan, fosfomycin is approved for the treatment of pneumonia and diarrhea in cattle, and the resistance rate of fosfomycin of *Escherichia coli* from cattle has been reported to be ranging from 0% to 20% [2,3,4,5,6,7] and that of *Salmonella* spp. to be around 0% [8,9,10]. As fosfomycin is not included in antimicrobials that are routinely tested in the Japanese Veterinary Antimicrobial Resistance Monitoring System (JVARM) [11], the resistance rates in bacteria from cattle on a nationwide scale has been unknown.

Four mechanisms for the acquisition of fosfomycin resistance are currently known. The first is the modification and inactivation of fosfomycin. The modification and inactivation of fosfomycin involves *fosA*, *fosB*, *fosC* and *fosX* genes. The second is the extrusion of fosfomycin by efflux pumps. Efflux pumps that can expel fosfomycin are tet38, abaF (*Acinetobacter baumannii* Fosfomycin efflux), cusCFBA (Copper transporter) and mdtABC-TolC (multidrug transporter) [12]. Efflux pumps contribute to the phenotype of antibiotic resistance at different levels, depending on their expression level [13]. Third is the mutation of the enzyme that fosfomycin targets, specifically the enzyme murA [14]. Fourth is the decrease in the permeability of fosfomycin into the bacterial cell [12,15]. The decrease in permeability is mainly related to mutations in the glpT and uhpT transporters. It was observed that loss of uhpT alone results in a rise in MIC from 0.5 to 32, while loss of glpT caused an increase from 0.5 to 4, and loss of both leads to leap in MIC from 0.5 to >1024 [16]. It is also revealed that uhpA, uhpB and uhpC control the expression level of uhpT. In addition, cyaA and ptsI control expression levels of glpT and uhpT via cAMP expression. Mutations in these regulators can contribute to fosfomycin resistance [17,18,19].

Veterinary antimicrobial products are essential for treating bacterial infectious diseases in animals and consequently ensuring a stable supply of livestock and aquatic products. However, their inappropriate use can lead to the emergence of antimicrobial-resistant bacteria, posing risks to livestock, pets, aquatic animals, and humans. In Japan, the National Action Plan on Antimicrobial Resistance, created in 2016, has implemented numerous measures and is now in its second phase (2023–2027), promoting collaboration between human and veterinary medicine to combat antimicrobial resistance (AMR). One of the goals and strategies in the six areas of countermeasures on antimicrobial resistance outlined in the plan is to continuously monitor antimicrobial resistance and use of antimicrobials and to appropriately understand the signs of change and spread of antimicrobial resistance [20]. This research contributes to this by enhancing the understanding of fosfomycin resistance spread in the veterinary field.

This is the first report that investigates the resistance rate of fosfomycin in bacteria from cattle at the national level in Japan covering the years 2017, 2020, 2021 and 2022, in conjunction with whole-genome sequence analysis of fosfomycin resistance strains.

## 2. Results

### 2.1. Antimicrobial Susceptibility Testing

#### Minimum Inhibitory Concentration (MIC) Distributions and Resistance Rate

Antimicrobial susceptibility testing results for fosfomycin are summarized in Table 1. With the breakpoint of 256 μg/mL, the resistance rate to fosfomycin was 0.3% (95% CI [0–1.9%]) for *E. coli* derived from healthy cattle, 6.8% (95% CI [2.3–15.3%]) for *E. coli* derived from diseased cattle and 1.4% (95% CI [0–7.3%]) for *Salmonella* spp. derived from diseased cattle. In the resistance rate of *E. coli* from healthy cattle during the 4-year period (2017, 2020, 2021 and 2022), no difference was found at a significance level of 0.05 (*p* value = 0.39) by a χ square test. A Fisher exact test showed no differences in resistance rates in *E. coli* from diseased cattle between 2021 and 2022 at a significance level of 0.05 (*p* value = 0.2), nor in *Salmonella* spp. (*p* value = 1.0). These results suggest that resistance rates did not change among the years studied.

### 2.2. Whole-Genome Sequences

#### 2.2.1. In Silico Typing

WGS was performed on seven strains found to be resistant to fosfomycin, and in silico typing was conducted. ST type, O type, H type, phylotype and serotype of strains are presented in Table 2 with MIC values.

#### 2.2.2. Resistance Genes

The results of detecting antimicrobial resistance genes are shown in Table 3 (AMR gene names and corresponding resistant antimicrobial agents are shown in Supplemental Table S1). The results of antimicrobial susceptibility testing (AST) conducted in JVARM are also indicated in Table 3. JVARM routinely tests ampicillin, cefazolin, cefotaxime, chloramphenicol, ciprofloxacin, colistin, gentamicin, kanamycin, meropenem, nalidixic acid, streptomycin, sulfamethoxazole and trimethoprim, and tetracycline. Five out of the six fosfomycin-resistant *E. coli* strains carried *fosA3*. The strain from healthy cattle did not carry resistance genes. The fosfomycin-resistant *Salmonella* strain carried *fosA3* and *fosA7*. Resistance genes to other antimicrobials were also detected. Six *sul2* genes, five *floR* genes and four *aph(3″)-Ib*, *aph(6)-Id*, *bla*_CTX-M_, *bla*_TEM_ and *tet*(A) genes were found among strains alongside *fosA3*. Co-location of resistance genes with *fosA*-group genes were also checked. In the *Salmonella* strain, *fosA3* was placed in the same contig as *bla*_CTX-M-14_. We also checked whether the contigs where *fosA*-group genes reside are chromosome or plasmid. Four out of seven contigs with *fosA*-group genes were predicted as plasmids.

#### 2.2.3. Efflux Pumps

The gene sequences of fosfomycin-resistant strains were annotated, and the pre-annotated genbank file of ATCC^®^ 25922 was obtained from the Nucleotide database [21]. The genes found are shown in Table 4. The *cusCFBA* was present in all *E. coli* strains including ATCC^®^ 25922. The *abaF* and *mdtABC-TolC* were present in all the fosfomycin-resistant strains. No strains harbored *tet38*.

#### 2.2.4. Mutations

Using *E. coli* ATCC^®^ 25922 (known as being susceptible to fosfomycin) as the reference sequence, we compared the sequences of fosfomycin-resistant *E. coli* strains to detect mutations. The results are shown in Table 5. Sequences of the gene encoding enzyme that fosfomycin targets (*murA*), transporters where fosfomycin enters into the bacterial cell (*glpT* and *uhpT*) and regulators of the transporters (*uhpA*, *uhpB*, *uhpC*, *cyaA* and *ptsI*) were investigated. *uhpB* and *cyaA* were not detected in the reference sequence. Therefore, analyses of SNPs were conducted only in *murA*, *glpT*, *uhpT*, *uhpC*, *uhpA* and *ptsI* genes. Synonymous mutations are excluded from Table 5 because their putative impact is low. There were mutations detected in *uhpA* but the type of mutations were all synonymous mutations. One strain of *E. coli* from diseased cattle has a missense mutation in *murA* (c.295C>T p.Pro99Ser). Among transporter genes, the sequence from *E. coli* derived from healthy cattle had a missense mutation (c.559_561delCACinsTAT p.His187Tyr) in *glpT* and a stop gained mutation (c.409C>T p.Gln137*) in *uhpT*. From diseased cattle, a missense mutation (c.1342A>G p.Lys448Glu) and a stop gained mutation (c.545G>A p.Trp182*) were found in *glpT*. Seven mutation variations were detected in *uhpC*, and all were missense mutations. Amongst the *uhpC* mutations, c.1249_1251delGCCinsTCT p.Ala417Ser was detected the most frequently and found in six strains. The detected missense mutations in *ptsI* included three variations that were c.916A>G p.Thr306Ala (four strains), c.916_918delACGinsGCA p.Thr306Ala (two strains) and c.1100A>G p.Lys367Arg (two strains).

## 3. Discussion

*Escherichia coli* strains were sampled from healthy cattle (*n* = 292, combined total from 2017, 2020, 2021 and 2022) and diseased cattle (*n* = 73, from 2021 to 2022). *Salmonella* spp. samples were obtained from diseased cattle (*n* = 74 from 2021 to 2022). These samples originated from different and non-duplicated farms. The resistance rates to fosfomycin in *E. coli* from healthy cattle (0.3% (95% CI [0–1.9%])), *E. coli* (6.8% (95% CI [2.3–15.3%])) and *Salmonella* spp. (1.4% (95% CI [0–7.3%])) from diseased cattle in Japan were relatively lower than those of other antimicrobial agents. For example, in the 2022 samples taken from healthy cattle, the resistance rates of *E. coli* for tetracycline, streptomycin and chloramphenicol (these are the top three in the resistance rates) were 23.4%, 18.5% and 7.3%, respectively, and those from diseased cattle were 66.7%, 61.8% and 51.0%. The resistance rates in *Salmonella* spp. from diseased cattle in 2022 against ampicillin, tetracycline and chloramphenicol were 55.4%, 53.0% and 41.0%, respectively [22]. Four drugs containing fosfomycin as the main active ingredient have been approved in Japan: three for cattle and one for *Perciformes*. In 2022, the total weight of veterinary antimicrobial agents sold for treatment was 777,759 kg in active ingredient form, and fosfomycin accounted for only 0.07% of that, indicating that fosfomycin is used infrequently [23]. The amount of fosfomycin used was less than other antimicrobial agents, which may explain why the resistance rate to fosfomycin was less than other antimicrobial agents.

In our study, *fosA3* was found in six out of the seven strains that were resistant to fosfomycin. It is reported that *fosA3* is the most spread variant in Enterobacterales, followed by *fosA7* and *fosA5* [24]. It is also reported that among the *fosA3*-positive *E. coli* strains from animals in Hong Kong, 95.9% harbored *bla*_CTX-M_ [25]. Another study in China found that *fosA3* was co-located with *bla*_CTX-M_ genes on conjugative plasmids in *Escherichia coli* strains from animals [26]. In our strains, four out of the six strains with *fosA3* had *bla*_CTX-M_ simultaneously (67%), and one *Salmonella* spp. strain showed the co-location of *fosA3* and *bla*_CTX-M-14_ on the same plasmid. These results are consistent with previous reports. However, it is important to note that one study in Japan reported that CTX-M-producing *E. coli* strains from human medical facilities that have acquired fosfomycin resistance are infrequent (3.6%) [27]. Regarding *Salmonella* spp., a high prevalence of strains that harbor at least one *fosA*-group fosfomycin resistance gene was reported, and the identified genes were *fosA7*, *fosA7.2*, *fosA3*, *fosA7.3 and fosA7.4* [28]. In this study, WGS was carried out using short-read sequences; however, analysis using long-read sequences is preferable to detect genes that reside with *fosA* genes in addition to plasmids, as short reads hardly yield complete circular plasmid sequences. In Japan, it has been found that 30% of *Salmonella* serotypes isolated from cattle in 2011–2021 were of the Typhimurium variant (4:i:-), 27% were Typhimurium and 5% were Dublin [11,29]. In this study, the *Salmonella* serotype found to be resistant to fosfomycin was Montevideo, which is a minor serotype.

*CusCFBA* was present in ATCC^®^ 25922, though ATCC^®^ 25922 is reported to have an MIC of 0.5–2 μg/mL [20]. This indicates that the existence of this transporter alone does not affect the resistant phenotype to fosfomycin. Since efflux pumps can contribute to the phenotype of antibiotic resistance at different levels, depending on their expression level [13], we need further techniques like qPCR [30] or RNA-seq [31] to directly measure overexpression. In the analysis of mutations, we observed mutations both in *glpT* and *uhpT* in the strains from healthy cattle. This strain did not possess *fosA*-group genes. A study shows that loss of both *glpT* and *uhpT* genes leads to resistance to fosfomycin [16], so these strains may have acquired fosfomycin resistance from the mutations we observed. However, since we did not examine the functionality of the proteins, it is premature to conclude causality. Also, a missense mutation of the *murA* gene was found in one strain, and the mutation is different from mutations reported previously [32,33,34,35,36]. This may cause the affinity with fosfomycin to decline; however, we need further analysis to conclude the effect on the functionality of the murA protein that is gained from the mutated gene.

## 4. Materials and Methods

### 4.1. Strains Collection

The strains used in this study were collected by JVARM using their standard procedures. The target number of *E. coli* strains from healthy cattle per year was set at 73, and strains were collected in 2017, 2020, 2021 and 2022, totaling 292 strains. This number was calculated based on the assumption that the resistance rate to fosfomycin is 5%, and we wanted to obtain results within 5% accuracy (95% confidence). The target number of strains of *E. coli* and *Salmonella* spp. from diseased cattle per year was set at 37, and strains were collected in 2021 and 2022, reaching a total of 74 strains for each species. This number was calculated by estimating that the resistance rate is 5% and the desired accuracy was within 7% with 95% confidence. The 2022 count of *E. coli* strains from diseased cattle was 36, because WGS revealed that one strain was not *Escherichia coli* and it was excluded. The strains used were randomly chosen from JVARM’s strain collection. We confirmed that the chosen samples originated from different and non-duplicated farms.

In JVARM, strains from healthy livestock were obtained by sampling the rectum stool from cattle in slaughterhouses. Strains from diseased cattle were collected from samples taken for pathological inspection conducted by livestock hygiene service centers across the country. The samples for pathological inspection were taken from different organs (liver, lung, intestinal contents, etc.) and various diseases (diarrhea, sudden death, sepsis, etc.). The broth microdilution method proposed by the Clinical and Laboratory Standards Society (CLSI) was performed in JVARM [37].

### 4.2. Antimicrobial Susceptibility Testing

An agar dilution method was performed according to CLSI M07 11th ed. [38] to measure the MICs of fosfomycin. The concentration range of fosfomycin was set at 0.25–512 μg/mL. Glucose-6-Phosphate was added as an additive to a final concentration of 25 μg/mL. As quality control strains, *E. coli* ATCC^®^ 25922 and *Enterococcus faecalis* ATCC^®^ 29212 were used. The breakpoint was set at 256 μg/mL or higher based on CLSI M100 33rd ed [39].

### 4.3. Whole-Genome Sequence

Whole-genome sequencing was performed on the strains that showed MICs larger or equal to 256 μg/mL to fosfomycin. DNA isolates were prepared for WGS using the DNeasy Blood and Tissue Kit (Qiagen, Valencia, CA, USA). Tagmentation and PCR amplification were performed using a Nextera XT DNA Isolate Preparation Kit (Illumina, Inc., San Diego, CA, USA). WGS was carried out using a MiSeq instrument (Illumina, Inc., San Diego, CA, USA) in paired-end mode (2 × 300). Quality control and trimming of MiSeq raw reads were performed with fastp v0.23.4 [40]. De novo assembly was performed using Shovill v1.1.0 [41].

- In Silico Typing

ST type was determined by fastMLST v0.0.16 [42]. For *E. coli*, O and H typing was conducted by using Ectyper v1.0.0 [43] and phylotype typing by ezclermont v0.7.0 [44], and for *Salmonella* spp., O and H and *salmonella* typing was analyzed by SISTR v1.1.2 [45].

- Resistance Genes

AMRFinderPlus v3.12.8 [46] was used to detect AMR genes among contigs. MOB-suite v3.1.9 [47] was used to predict whether each contig was a chromosome or a plasmid.

- Efflux Genes

Prokka v1.14.6 [48] was used to annotate genes and Biopython v1.84 [49] was used to check the existence of genes interested (*abaF*, *cusCFBA*, *mdtABC-TolC* and *tet38*) in the annotated sequences.

- Mutations

The genomic sequence of *E. coli* ATCC^®^ 25922 was obtained from the Nucleotide database, National Library of Medicine (Accession No. NZ_CP009072) as a Genbank file format [21]. Then, this sequence was used as the reference sequence, and Snippy v4.6.0 [50] was used to detect mutations among the strain sequences.

- Statistical Analyses

A chi-square test and Fisher’s exact test were performed by SciPy v1.11.1 [51], and data wrangling was performed using pandas v1.5.3 [52].

## 5. Conclusions

In this study, the nationwide scale of resistance rates to fosfomycin in *E. coli* isolated from healthy and diseased cattle and that of *Salmonella* spp. from diseased cattle were revealed for the first time, and the resistance rates were low. Fosfomycin resistance strains have *fosA3* except one strain and four out of seven *fosA*-group genes were found on plasmids. Besides the existence of *fosA*-group genes, several mechanisms that may confer fosfomycin resistance were identified in the resistant strains. Since fosfomycin is regarded as one of the highest-priority critically important antimicrobials for human medicine on the WHO’s list, we should keep the resistance rate to fosfomycin low in the veterinary field in accordance with the one health approach.

## Figures and Tables

**Table 1:** Fosfomycin MIC distribution table. Vertical line shows the breakpoint.

Origin	Species	Year	MIC 50	MIC 90	%R [95% CI]	*n*	Number of Strains with MIC (μg/mL) of												
							≤0.25	0.5	1	2	4	8	16	32	64	128	256	512	>512
Healthy cattle	*E. coli*	2017	1	4	0.0 [0.0–4.9]	73	0	11	34	17	5	2	3	1	0	0	0	0	0
		2020	1	4	0.0 [0.0–4.9]	73	0	7	38	17	8	0	1	2	0	0	0	0	0
		2021	1	4	1.4 [0.0–7.4]	73	0	4	49	10	4	2	0	2	1	0	1	0	0
		2022	1	4	0.0 [0.0–4.9]	73	0	2	42	17	9	0	1	2	0	0	0	0	0
		Total	1	4	0.3 [0.0–1.9]	292	0	24	163	61	26	4	5	7	1	0	1	0	0
Diseased cattle	*E. coli*	2021	1	4	2.7 [0.1–14.2]	37	0	15	14	4	1	0	1	1	0	0	0	0	1
		2022 *	1	<512	11.1 [3.1–26.1]	36	0	2	19	4	1	2	1	2	1	0	0	0	4
		Total	1	32	6.8 [2.3–15.3]	73	0	17	33	8	2	2	2	3	1	0	0	0	5
	*Salmonella* spp.	2021	0.5	0.5	0.0 [0.0–9.5]	37	18	18	0	1	0	0	0	0	0	0	0	0	0
		2022	0.5	1	2.7 [0.1–14.2]	37	5	28	1	1	0	0	1	0	0	0	0	1	0
		Total	0.5	0.5	1.4 [0.0–7.3]	74	23	46	1	2	0	0	1	0	0	0	0	1	0

* One strain suspected as contamination was excluded.

**Table 2:** Profiles of seven strains that were resistant to fosfomycin in antimicrobial susceptibility testing.

Origin	Species	Strain No.	MIC	ST Type	O Type	H Type	Phylotype	Serotype
Healthy cattle	*E. coli*	E-1	256	300	O76	H25	B1	-
Diseased cattle	*E. coli*	E-2	>512	21	O26	H11	B1	-
	*E. coli*	E-3	>512	744	O89	H9	A	-
	*E. coli*	E-4	>512	10	O9	H9	A	-
	*E. coli*	E-5	>512	16	O119	H8	B1	-
	*E. coli*	E-6	>512	162	O9	H9	B1	-
	*Salmonella* spp.	S-1	512	138	6, 7, 14 [54]	g, m, s	-	Montevideo

**Table 3:** Detected resistant genes in the strains resistant to fosfomycin.

Origin	Strain No.	Resistance Phenotype by AST Result ^a^	AMR Genes		Resistance Gene in the Contig with *fosA*	Type of the Contig with *fosA*	Nearest Plasmid ^b^
			Fosfomycin	Other Antimicrobials			
Healthy cattle	E-1	**-**	**-**	**-**	-	-	
Diseased cattle	E-2	ABPC, CEZ, CTX	*fosA3*	*bla*_CTX-M-3_, *bla*_TEM_, *sul2*	-	chromosome	
	E-3	ABPC, CEZ, CP, CPFX, CTX, KM, NA, SM, TC	*fosA3*	*aph(3″)-Ib*, *aph(3′)-Ia*, *aph(6)-Id*, *bla*_CTX-M-55_, *bla*_TEM_, *floR*, *sul2*, *tet*(A)	-	plasmid	p13P484A-2
	E-4	ABPC, CP, CPFX, GM, NA, SM, ST, TC	*fosA3*	*aac(3)-IId*, *aph(3″)-Ib*, *aph(6)-Id*, *bla*_TEM-1_, *dfrA14*, *floR*, *sul2*, *tet*(A)	-	chromosome	
	E-5	CP, KM, NA, SM, ST, TC	*fosA3*	*aadA2*, *aph(3″)-Ib*, *aph(6)-Id*, *dfrA12*, *floR*, *mph(A)*, *qacEdelta1*, *sul1*, *sul2*, *tet*(A)	-	plasmid	pSF07201
	E-6	ABPC, CEZ, CP, CPFX, CTX, NA, SM, ST, TC	*fosA3*	*aph(3″)-Ib*, *aph(6)-Id*, *bla*_CTX-M-55_, *bla*_TEM_, *dfrA14*, *floR*, *sul2*, *tet*(A)	-	plasmid	pHNAH17
	S-1	ABPC, CEZ, CP, CTX, GM, KM, SM	*fosA3*	*aac(3)-IVa*, *aadA1*, *aadA2*, *aph(3′)-Ia*, *aph(4)-Ia*, *bla*_CTX-M-14_, *cmlA1*, *floR*, *mcr-1*, *qacL*, *sul2*, *sul3*	*bla*_CTX-M-14_	plasmid	pLS44712-MCR
			*fosA7*		-	chromosome	

^a^ Abbreviations used in AST result: ampicillin (ABPC), cefazolin (CEZ), chloramphenicol (CP), ciprofloxacin (CPFX), cefotaxime (CTX), gentamicin (GM), kanamycin (KM), nalidixic acid (NA), streptomycin (SM), sulfamethoxazole and trimethoprim (ST), and tetracycline (TC). A microdilution method was conducted following CLSI guidelines. ^b^ The nearest neighbor plasmids to the *fosA*-containing contigs were determined by MOB-suite.

**Table 4:** Detected efflux genes in the strains. (+ indicates presence and − indicates absence of the gene).

Origin	Strain No.	*abaF*				*cusCFBA*							*mdtABC-tolC*							
		*abaF*	*abaF_1*	*abaF_2*		*cusA*	*cusB*	*cusC*	*cusC_1*	*cusC_2*	*cusF*		*mdtA*	*mdtB*	*mdtC*	*tolC*	*tolC_1*	*tolC_2*		*tet38*
ATCC^®^ 25922	−	−	−	−		+	+	+	−	−	+		−	−	−	+	−	−		−
Healthy cattle	E-1	−	+	+		+	+	−	+	+	+		+	+	+	+	−	−		−
Diseased cattle	E-2	+	−	−		+	+	−	+	+	+		+	+	+	+	−	−		−
	E-3	+	−	−		+	+	−	+	+	+		+	+	+	+	−	−		−
	E-4	+	−	−		+	+	−	+	+	+		+	+	+	+	−	−		−
	E-5	+	−	−		+	+	−	+	+	+		+	+	+	+	−	−		−
	E-6	+	−	−		+	+	−	+	+	+		+	+	+	+	−	−		−
	S-1	+	−	−		+	−	−	−	−	−		+	+	+	−	+	+		−

**Table 5:** Mutations in sequences from fosfomycin-resistant bacteria.

Origin	Strain No.	*murA*	*glpT*		*uhpT*	*uhpC*	*ptsI*
		Missense	Missense	Stop Gained	Stop Gained	Missense	Missense
Healthy cattle	E-1	-	- c.559_561delCACinsTAT p.His187Tyr	-	- c.409C>T p.Gln137*	- c.127T>G p.Tyr43Asp - c.529_531delAGCinsGCT p.Ser177Ala - c.1249_1251delGCCinsTCT p.Ala417Ser - c.1303A>G p.Thr435Ala	- c.916A>G p.Thr306Ala
Diseased cattle	E-2	-	-	c.545G>A p.Trp182*	-	- c.52C>T p.His18Tyr - c.321_324delTATCinsCATG p.Ile108Met - c.1249_1251delGCCinsTCT p.Ala417Ser - c.1264A>G p.Ile422Val - c.1303A>G p.Thr435Ala	- c.916A>G p.Thr306Ala
	E-3	-	-	-	-	- c.52C>T p.His18Tyr - c.529_531delAGCinsGCT p.Ser177Ala - c.1249_1251delGCCinsTCT p.Ala417Ser	- c.916_918delACGinsGCA p.Thr306Ala - c.1100A>G p.Lys367Arg
	E-4	- c.295C>T p.Pro99Ser	- c.1342A>G p.Lys448Glu	-	-	- c.52C>T p.His18Tyr - c.529_531delAGCinsGCT p.Ser177Ala - c.1249_1251delGCCinsTCT p.Ala417Ser	- c.916_918delACGinsGCA p.Thr306Ala - c.1100A>G p.Lys367Arg
	E-5	-	-	-	-	- c.52C>T p.His18Tyr - c.321_324delTATCinsCATG p.Ile108Met - c.1249_1251delGCCinsTCT p.Ala417Ser - c.1264A>G p.Ile422Val - c.1303A>G p.Thr435Ala	- c.916A>G p.Thr306Ala
	E-6	-	-	-	-	- c.529_531delAGCinsGCT p.Ser177Ala - c.1249_1251delGCCinsTCT p.Ala417Ser - c.1303A>G p.Thr435Ala	- c.916A>G p.Thr306Ala

## Data Availability

All raw short-read sequence data have been deposited in the DNA Data Bank of Japan (BioProject: PRJDB19617).

## Supplementary Materials

The following supporting information can be downloaded at: https://www.mdpi.com/article/10.3390/ijms252413723/s1.

## References

[1] World Health Organization WHO’s List of Medically Important Antimicrobials: A Risk Management Tool for Mitigating Antimicrobial Resistance Due to Non-Human Use. https://cdn.who.int/media/docs/default-source/gcp/who-mia-list-2024-lv.pdf.

[2] Maehara T., Kita T., Fujino Y., Tsujimoto M. (2005). Prevalence and Drug-susceptibility of Enterohemorrhagic *Escherichia coli* O157 in Cattle during the Summer. J. Jpn. Vet. Med. Assoc..

[3] Miyane K. (2021). Antibiotic resistance investigation of bacterial pathogens isolated from cattle in Tokachi area, Hokkaido from 2010 to 2018. J. Farm Anim. Infect. Dis..

[4] Abe Y., Watanabe T., Honda M. Comparison of Antimicrobial-Resistant Enterohemorrhagic Escherichia coli (EHEC) Isolated from Humans and Cattles. https://www.city.fukuoka.lg.jp/hokanken/kenkyu/shohou/documents/SHOHOU.pdf.

[5] Matayoshi M. (2010). Antimicrobial Susceptibility and Resistance Genes Among Enterotoxigenic *Escherichia coli* and ShigatoxinhProducing *Escherichia coli* Isolated from Diarrheic Calves, in Okinawa Prefecture. J. Jpn. Vet. Med. Assoc..

[6] Nakamura Y., Kawase J., Suga M., Fujita Y., Murakami Y., Kawakami Y., Tabara K., Hirata A. (2016). Prevalence and Molecular Epidemiological Analysis of Enterohemorrhagic *Escherichia coli* in Cattle at a Slaughterhouse in Shimane Prefecture, Japan. J. Jpn. Vet. Med. Assoc..

[7] Asoshima N., Matsuda M., Honda M., Shinohara T., Hiwaki H. (2012). Characterization of Extended-Spectrum β-Lactamase-Producing *Escherichia coli* Strains Isolated from Domestic Animals, Chicken Meat and Humans. Jpn. J. Food Microbiol..

[8] Ido N., Kudo T., Sasaki K., Motokawa M., Iwabuchi K., Matsudate H., Seimiya Y.M., Akiba M. (2011). Molecular and phenotypic characteristics of *Salmonella enterica* serovar 4,5,12:i:- isolated from cattle and humans in Iwate Prefecture, Japan. J. Vet. Med. Sci..

[9] Ido N., Lee K., Iwabuchi K., Izumiya H., Uchida I., Kusumoto M., Iwata T., Ohnishi M., Akiba M. (2014). Characteristics of *Salmonella enterica* serovar 4,(5),12:i:- as a monophasic variant of serovar Typhimurium. PLoS ONE.

[10] Arai N., Sekizuka T., Tamamura-Andoh Y., Barco L., Hinenoya A., Yamasaki S., Iwata T., Watanabe-Yanai A., Kuroda M., Akiba M. (2021). Identification of a Recently Dominant Sublineage in *Salmonella* 4,(5),12:i:- Sequence Type 34 Isolated From Food Animals in Japan. Front. Microbiol..

[11] National Veterinary Assay Laboratory, Ministry of Agriculture, Forestry and Fisheries of Japan Report on the Japanese Veterinary Antimicrobial Resistance Monitoring System 2018–2019. https://www.maff.go.jp/nval/yakuzai/pdf/JVARM_Report_2018-2019.pdf.

[12] Wangchinda W., Rattanaumpawan P. (2022). JMM Profile: Fosfomycin: A potential antibiotic for multi- and extensively resistant bacteria. J. Med. Microbiol..

[13] Blanco P., Hernando-Amado S., Reales-Calderon J.A., Corona F., Lira F., Alcalde-Rico M., Bernardini A., Sanchez M.B., Martinez J.L. (2016). Bacterial Multidrug Efflux Pumps: Much More Than Antibiotic Resistance Determinants. Microorganisms.

[14] Fu Z., Ma Y., Chen C., Guo Y., Hu F., Liu Y., Xu X., Wang M. (2015). Prevalence of Fosfomycin Resistance and Mutations in *murA*, *glpT*, and *uhpT* in Methicillin-Resistant *Staphylococcus aureus* Strains Isolated from Blood and Cerebrospinal Fluid Samples. Front. Microbiol..

[15] Castaneda-Garcia A., Blazquez J., Rodriguez-Rojas A. (2013). Molecular Mechanisms and Clinical Impact of Acquired and Intrinsic Fosfomycin Resistance. Antibiotics.

[16] Xu S., Fu Z., Zhou Y., Liu Y., Xu X., Wang M. (2017). Mutations of the Transporter Proteins GlpT and UhpT Confer Fosfomycin Resistance in *Staphylococcus aureus*. Front. Microbiol..

[17] Cattoir V., Pourbaix A., Magnan M., Chau F., de Lastours V., Felden B., Fantin B., Guerin F. (2020). Novel Chromosomal Mutations Responsible for Fosfomycin Resistance in *Escherichia coli*. Front. Microbiol..

[18] Cattoir V., Guerin F. (2018). How is fosfomycin resistance developed in *Escherichia coli*?. Future Microbiol..

[19] Li X., Quan J., Yang Y., Ji J., Liu L., Fu Y., Hua X., Chen Y., Pi B., Jiang Y. (2016). Abrp, a new gene, confers reduced susceptibility to tetracycline, glycylcine, chloramphenicol and fosfomycin classes in *Acinetobacter baumannii*. Eur. J. Clin. Microbiol. Infect. Dis..

[20] The Government of Japan National Action Plan on Antimicrobial Resistance (AMR) 2023–2027. https://www.mhlw.go.jp/content/10900000/001096228.pdf.

[21] Minogue T.D., Daligault H.A., Davenport K.W., Bishop-Lilly K.A., Broomall S.M., Bruce D.C., Chain P.S., Chertkov O., Coyne S.R., Freitas T. (2014). Complete Genome Assembly of *Escherichia coli* ATCC 25922, a Serotype O6 Reference Strain. Genome Announc..

[22] National Veterinary Assay Laboratory, Ministry of Agriculture, Forestry and Fisheries of Japan Resistance Rates Data from JVARM Results. https://www.maff.go.jp/nval/yakuzai/yakuzai_AMR_2.html.

[23] National Veterinary Assay Laboratory, Ministry of Agriculture, Forestry and Fisheries of Japan Sales Data of Antimicrobial Agents from JVARM. https://www.maff.go.jp/nval/yakuzai/yakuzai_p3_6.html.

[24] Mattioni Marchetti V., Hrabak J., Bitar I. (2023). Fosfomycin resistance mechanisms in Enterobacterales: An increasing threat. Front. Cell Infect Microbiol..

[25] Chan J., Lo W.U., Chow K.H., Lai E.L., Law P.Y., Ho P.L. (2014). Clonal diversity of *Escherichia coli* isolates carrying plasmid-mediated fosfomycin resistance gene *fosA3* from livestock and other animals. Antimicrob. Agents Chemother..

[26] Wang X.M., Dong Z., Schwarz S., Zhu Y., Hua X., Zhang Y., Liu S., Zhang W.J. (2017). Plasmids of Diverse Inc Groups Disseminate the Fosfomycin Resistance Gene fosA3 among *Escherichia coli* Isolates from Pigs, Chickens, and Dairy Cows in Northeast China. Antimicrob. Agents Chemother..

[27] Wachino J., Yamane K., Suzuki S., Kimura K., Arakawa Y. (2010). Prevalence of fosfomycin resistance among CTX-M-producing *Escherichia coli* clinical isolates in Japan and identification of novel plasmid-mediated fosfomycin-modifying enzymes. Antimicrob. Agents Chemother..

[28] Monte D.F.M., Doi Y., Lincopan N. (2023). High prevalence and global distribution of fosfomycin resistance genes in *Salmonella* serovars. Lancet Microbe.

[29] The AMR One Health Surveillance Committee Nippon AMR One Health Report (NAOR) 2023. https://www.mhlw.go.jp/content/10900000/001268945.pdf.

[30] Abd El-Rahman O.A., Rasslan F., Hassan S.S., Ashour H.M., Wasfi R. (2023). The RND Efflux Pump Gene Expression in the Biofilm Formation of *Acinetobacter baumannii*. Antibiotics.

[31] Lee J.J., Kang H.Y., Lee W.I., Cho S.Y., Kim Y.J., Lee H.J. (2021). Efflux pump gene expression study using RNA-seq in multidrug-resistant TB. Int. J. Tuberc. Lung Dis..

[32] Xin L., Hu Z., Han R., Xu X., Wang C., Li D., Guo Y., Hu F. (2022). Asp50Glu mutation in MurA results in fosfomycin resistance in Enterococcus faecium. J. Glob. Antimicrob. Resist..

[33] Takahata S., Ida T., Hiraishi T., Sakakibara S., Maebashi K., Terada S., Muratani T., Matsumoto T., Nakahama C., Tomono K. (2010). Molecular mechanisms of fosfomycin resistance in clinical isolates of Escherichia coli. Int. J. Antimicrob. Agents.

[34] Cheng G., Hu Y., Lu N., Li J., Wang Z., Chen Q., Zhu B. (2013). Identification of a novel fosfomycin-resistant UDP-N-acetylglucosamine enolpyruvyl transferase (MurA) from a soil metagenome. Biotechnol. Lett..

[35] Kim D.H., Lees W.J., Kempsell K.E., Lane W.S., Duncan K., Walsh C.T. (1996). Characterization of a Cys115 to Asp substitution in the Escherichia coli cell wall biosynthetic enzyme UDP-GlcNAc enolpyruvyl transferase (MurA) that confers resistance to inactivation by the antibiotic fosfomycin. Biochemistry.

[36] Wanke C., Amrhein N. (1993). Evidence that the reaction of the UDP-N-acetylglucosamine 1-carboxyvinyltransferase proceeds through the O-phosphothioketal of pyruvic acid bound to Cys115 of the enzyme. Eur. J. Biochem..

[37] National Veterinary Assay Laboratory, Ministry of Agriculture, Forestry and Fisheries of Japan Japanese Veterinary Antimicrobial Resistance Monitoring Annual Report. https://www.maff.go.jp/nval/yakuzai/pdf/Annual_Report_2020.pdf.

[38] CLSI (2018). Methods for Dilution Antimicrobial Susceptibility Tests for Bacteria That Grow Aerobically.

[39] CLSI (2023). Performance Standards for Antimicrobial Susceptibility Testing.

[40] Chen S. (2023). Ultrafast one-pass FASTQ data preprocessing, quality control, and deduplication using fastp. Imeta.

[41] Seemann T. Shovill: Assemble Bacterial Isolate Genomes from Illumina Paired-End Reads. https://github.com/tseemann/shovill.

[42] Guerrero-Araya E., Munoz M., Rodriguez C., Paredes-Sabja D. (2021). FastMLST: A Multi-core Tool for Multilocus Sequence Typing of Draft Genome Assemblies. Bioinform. Biol. Insights.

[43] Bessonov K., Laing C., Robertson J., Yong I., Ziebell K., Gannon V.P.J., Nichani A., Arya G., Nash J.H.E., Christianson S. (2021). ECTyper: In silico *Escherichia coli* serotype and species prediction from raw and assembled whole-genome sequence data. Microb. Genom..

[44] Waters N.R., Abram F., Brennan F., Holmes A., Pritchard L. (2020). Easy phylotyping of *Escherichia coli* via the EzClermont web app and command-line tool. Access Microbiol..

[45] Yoshida C.E., Kruczkiewicz P., Laing C.R., Lingohr E.J., Gannon V.P., Nash J.H., Taboada E.N. (2016). The *Salmonella* in Silico Typing Resource (SISTR): An Open Web-Accessible Tool for Rapidly Typing and Subtyping Draft Salmonella Genome Assemblies. PLoS ONE.

[46] Feldgarden M., Brover V., Gonzalez-Escalona N., Frye J.G., Haendiges J., Haft D.H., Hoffmann M., Pettengill J.B., Prasad A.B., Tillman G.E. (2021). AMRFinderPlus and the Reference Gene Catalog facilitate examination of the genomic links among antimicrobial resistance, stress response, and virulence. Sci. Rep..

[47] Robertson J., Nash J.H.E. (2018). MOB-suite: Software tools for clustering, reconstruction and typing of plasmids from draft assemblies. Microb. Genom..

[48] Seemann T. (2014). Prokka: Rapid prokaryotic genome annotation. Bioinformatics.

[49] Cock P.J., Antao T., Chang J.T., Chapman B.A., Cox C.J., Dalke A., Friedberg I., Hamelryck T., Kauff F., Wilczynski B. (2009). Biopython: Freely available Python tools for computational molecular biology and bioinformatics. Bioinformatics.

[50] Seemann T. Snippy: Rapid Haploid Variant Calling and Core SNP Phylogeny. https://github.com/tseemann/snippy.

[51] Virtanen P., Gommers R., Oliphant T.E., Haberland M., Reddy T., Cournapeau D., Burovski E., Peterson P., Weckesser W., Bright J. (2020). SciPy 1.0: Fundamental algorithms for scientific computing in Python. Nat. Methods.

[52] The Pandas Development Team (2023). pandas-dev/pandas: Pandas (v1.5.3). Zenodo.

