# Supplementary material for: The Prevalence and the Underlying Mechanisms of Fosfomycin Resistance of Escherichia coli and Salmonella spp. Among Cattle in Japan
Source: Int J Mol Sci. 2024 Dec 23;25(24):13723. doi: 10.3390/ijms252413723 (PMC11676364; doi:10.3390/ijms252413723)
Supplement: Supplementary file 1 [file ijms-25-13723-s001.zip › ijms-3364210-supplementary.pdf]

Supplemental Table S1. AMR Gene Names and Corresponding Resistant Antimicrobial agents.

AMR gene	Resistant Antimicrobial agents
aac(3)-IId	Gentamicin
aac(3)-IVa	Apramycin/gentamicin/tobramycin
aadA1	Streptomycin
aadA2	Streptomycin
aph(3'')-Ib	Streptomycin
aph(3')-Ia	Kanamycin
aph(4)-Ia	Hygromycin
aph(6)-Id	Streptomycin
blaCTX-M-3	Cephalosporin
blaCTX-M-14	Cephalosporin
blaCTX-M-55	Cephalosporin
blaTEM	Beta-lactam
blaTEM-1	Beta-lactam
cmlA1	Chloramphenicol
dfrA12	Trimethoprim
dfrA14	Trimethoprim
floR	Chloramphenicol/florfenicol
fosA3	Fosfomycin
fosA7	Fosfomycin
mcr-1	Colistin
mph(A)	Azithromycin/erythromycin/spiramycin/telithromycin
qacEdelta1	Quaternary ammonium
qacL	Quaternary ammonium
sul1	Sulfonamide
sul2	Sulfonamide
sul3	Sulfonamide
tet(A)	Tetracycline
